# Validation of continuous intraabdominal pressure measurement: feasibility and accuracy assessment using a capsular device in in-vivo studies

**DOI:** 10.1186/s13017-024-00553-8

**Published:** 2024-06-26

**Authors:** Dong-Ru Ho, Chi-Tung Cheng, Chun-Hsiang Ouyang, Wei-Cheng Lin, Chien-Hung Liao

**Affiliations:** 1 Department of Urology, Chang Gung Memorial Hospita ChiaYi, 8, west section of Jiapu Road, Puzi, Chiayi, Taiwan; 2 Department of Trauma and Emergency Surgery, Chang Gung Memorial Hospital Linkou, Chang Gung University, Taoyuan, Taiwan; 3 Department of Electrical Engineering, Chang Gung University, Taoyuan, Taiwan; 4 School of Medicine, National Tsing Hua University, Hsinchu, Taiwan

**Keywords:** Intraabdominal pressure, Abdominal compartment syndrome, Capsular sensor, Digital health

## Abstract

**Background:**

Monitoring Intraabdominal Pressure (IAP) is essential in critical care, as elevated IAP can lead to severe complications, including Abdominal Compartment Syndrome (ACS). Advances in technology, such as digital capsules, have opened new avenues for measuring IAP non-invasively. This study assesses the feasibility and effectiveness of using a capsular device for IAP measurement in an animal model.

**Method:**

In our controlled experiment, we anesthetized pigs and simulated elevated IAP conditions by infusing CO2 into the peritoneal cavity. We compared IAP measurements obtained from three different methods: an intravesical catheter (IAP_ivp_), a capsular device (IAP_dot_), and a direct peritoneal catheter (IAP_dir_). The data from these methods were analyzed to evaluate agreement and accuracy.

**Results:**

The capsular sensor (IAP_dot_) provided continuous and accurate detection of IAP over 144 h, with a total of 53,065,487 measurement triplets recorded. The correlation coefficient (R²) between IAP_dot_ and IAP_dir_ was excellent at 0.9241, demonstrating high agreement. Similarly, IAP_ivp_ and IAP_dir_ showed strong correlation with an R² of 0.9168.

**Conclusion:**

The use of capsular sensors for continuous and accurate assessment of IAP marks a significant advancement in the field of critical care monitoring. The high correlation between measurements from different locations and methods underscores the potential of capsular devices to transform clinical practices by providing reliable, non-invasive IAP monitoring.

## Background

Intraabdominal pressure (IAP) refers to the pressure within the abdominal cavity, which is the space that houses visceral organs, and IAP is an essential pressure from the core of the body which might affect the blood supply into abdominal visceral organs. This pressure is influenced by various factors, including abdominal muscle tone, the contents of the abdominal cavity, body posture, and the pathophysiological changes from various disorders [1, 2]. IAP monitoring is crucial in critical care, impacting outcomes in trauma, burn, surgery, and acute medical conditions [3–6]. Elevated IAP can lead to severe complications, including abdominal compartment syndrome (ACS), organ dysfunction, and increased morbidity and mortality. Because of the importance of this physical parameter, some experts advocate IAP should be considered a core vital sign in critically ill patients.

Traditional methods for monitoring IAP, such as intravesical pressure measurement techniques, are both accurate and widespread. This widely adopted method involves intermittent manual measurements of IAP by instilling a maximum of 25 mL of sterile saline into the bladder. IAP readings should be expressed in mmHg and are typically taken at end-expiration while the patient is in the supine position, ensuring that there are no contractions of the abdominal muscles. The transducer must be zeroed at the level of the midaxillary line crossing the iliac crest. This standardization of the measurement process has been endorsed by the World Society on Abdominal Compartment Syndrome (WSACS, http://www.wsacs.org/) [7].

Despite its accuracy, this method carries certain risks, including the potential for retrograde urinary tract contaminations due to the invasive nature of the catheter utilized. Accurate measurement also requires skilled personnel, a resource that is often limited in healthcare settings. Moreover, the nature of this technique makes continuous monitoring of IAP impractical. Additionally, the accuracy of these hydrostatic pressure-based measurements can be compromised by the patient’s postural and lying angles, which can introduce bias into the results. These limitations underscore the urgent need for the development and validation of new tools and techniques that enhance the accuracy, convenience, and safety of continuous IAP monitoring.

Capsular devices have become well-established tools for monitoring various physical parameters, notably for their role in intraluminal imaging and detection [8]. The acceptance of these devices primarily stems from their ability to comfortably monitor the gastrointestinal (GI) tract without causing discomfort to patients. Their efficacy and patient satisfaction have been demonstrated in numerous studies, highlighting their widespread endorsement among health providers.

The primary advantages of capsular devices include their minimally invasive nature, enhanced patient comfort, and the capability for remote evaluation of the GI tract. Besides image examination, numerous applications for other physical parameter measurement and biomedical applications were presented. These capsular sensing devices represented a significant advancement to offer novel alternatives for patients. They have been effectively applied in measuring other vital parameters such as core temperature [9], intraabdominal pressure [10], bowel motility [11], intraluminal gas content [12], and microbiome behavior [13]. This versatility allows for continuous and comprehensive monitoring, making capsular devices a significant step forward in non-invasive medical technology.

Moreover, these devices have been adapted for advanced applications such as targeted drug delivery [14] and therapeutic vibration stimulation [15], exhibiting their potential beyond basic diagnostics. In this study, we employed a capsular device designed to continuously detect intraabdominal pressure using a non-invasive, real-time method. We conducted a validation study to assess the accuracy, precision, and reliability of this innovative IAP monitoring tool in animal models, comparing its performance against traditional intravesical pressure measurement techniques.

## Methods

### Animal instrumentation

This study was conducted in strict adherence to national guidelines for ethical animal research and received approval from the local Institutional Ethics Committee on Animal Care and Use. Following overnight fasting, eight anesthetized and paralyzed pigs (mean body weight of 25 *±* 1.0 kg) were mechanically ventilated using a MATRX VIP 3000™ Veterinary Anesthesia Vaporizer. Ventilation settings included an oxygen concentration (FiO2) of 35%, a tidal volume (TV) of 9 mL/kg, an inspiration/expiration ratio of 1/2, and a positive end-expiratory pressure (PEEP) of 7 cmH2O. The respiratory rate was adjusted to maintain arterial CO2 partial pressure (PaCO2) between 35 and 45 mmHg, and these settings were consistent throughout the experiment. For continuous monitoring of blood pressure and biochemical analyses, a catheter was inserted into the femoral artery. Hydration was maintained via intravenous administration of normal saline at a rate of 1.5 mL·kg^-1·h^-1 through a femoral vein catheter. The pigs remained supine throughout the study.

**Fig. 1:**
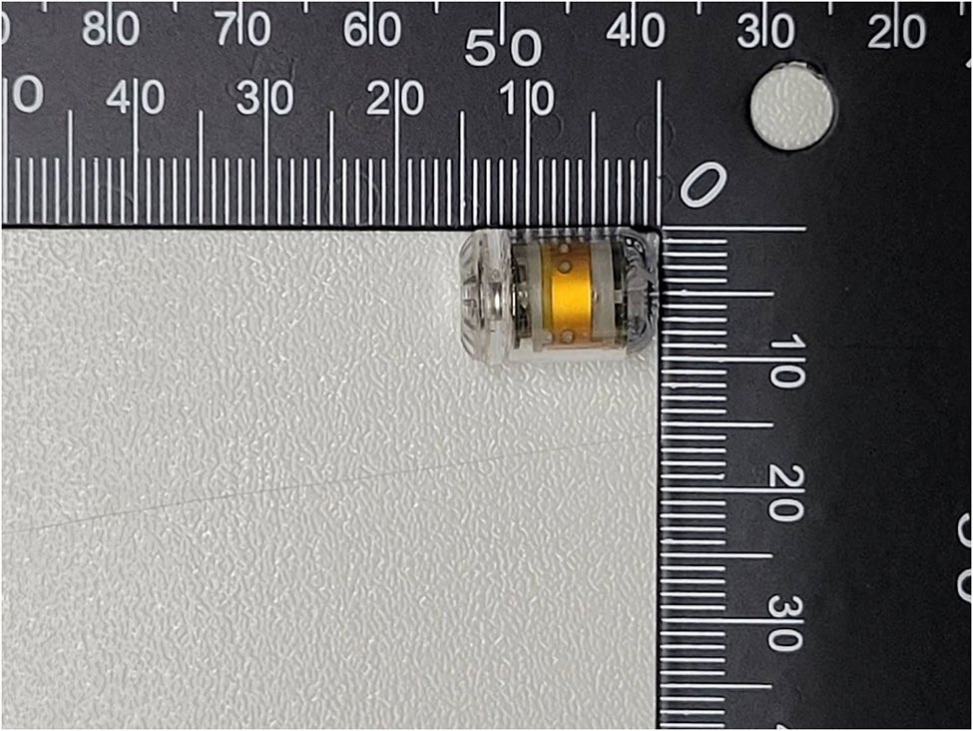
The swallowable capsular monitor device which used to detect intraabdominal pressure

The animals were instrumented with three different IAP measurement devices. The capsular pressure sensors: PressureDOT (Dotspace Inc., Delaware, United states) (IAP_dot_, Fig. 1) were positioned transesophageal into the stomach.

**Fig. 2:**
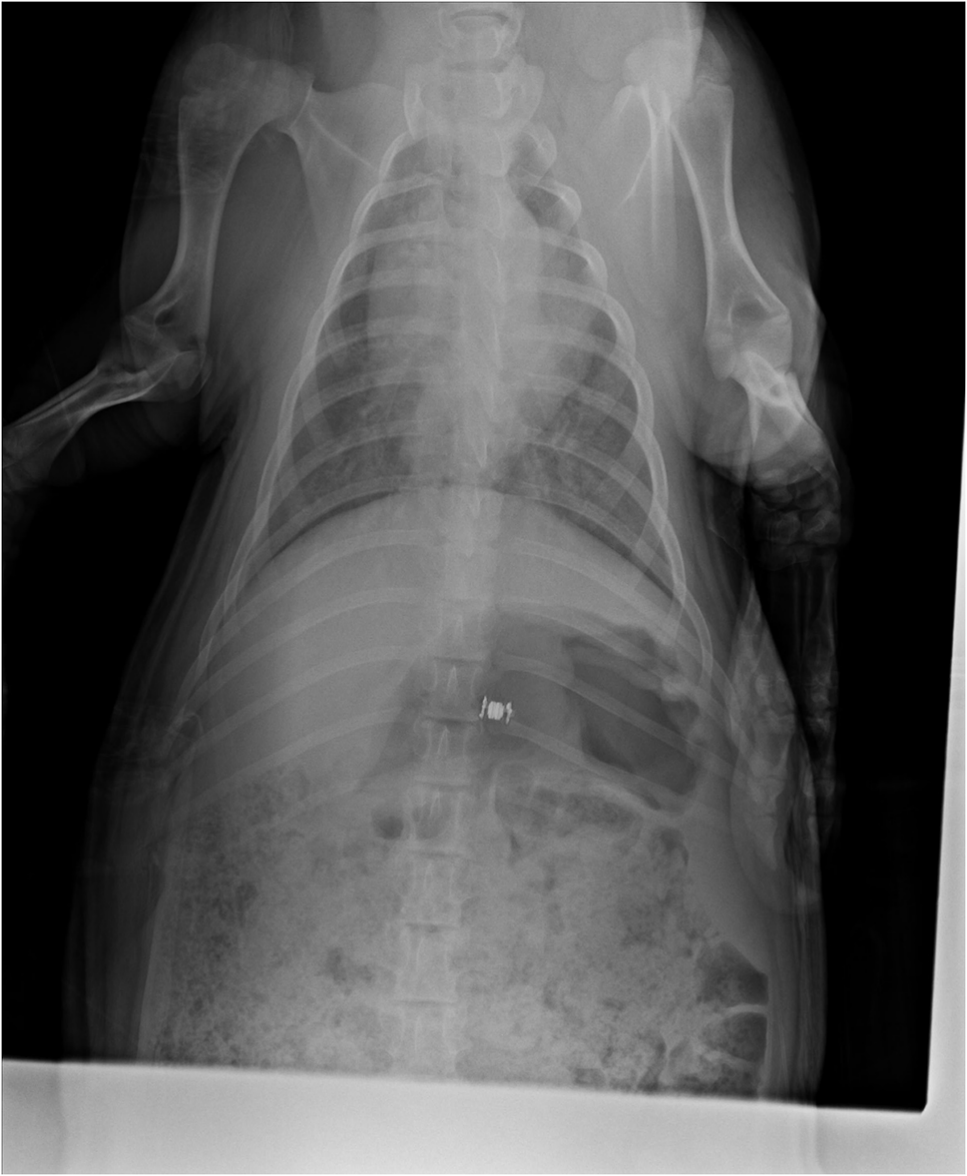
Radiographic imaging of a pig implanted with digital capsule for intraabdominal pressure monitoring

The position was checked afterwards by radiography. (as Fig. 2)

A small midline laparotomy was performed and a catheter (IAP_dir_) was placed intraperitoneally, caudally to the stomach. A Foley-based catheter with pressure transducer (IAP_ivp_) was inserted into the bladder. The two catheters were exteriorized, and the laparotomy was carefully closed and water-sealed in two layers.

### Measurements of IAP

Measurements of IAP were based on three different measurement principles.

#### PressureDOT capsular intraluminal IAP measurement (IAP_dot_)

The measurement of intra-abdominal pressure was conducted using a commercial medical device, the PressureDOT (Dotspace Inc., Delaware, United states). This device is an ingestible capsule (12 mm in length and 6 mm in diameter) equipped with temperature and pressure sensors. The device’s accuracy is ± 0.5 °C for temperature and ± 0.5 mmHg for pressure. It has a battery life of 300 h and transmits data every 5 s via Bluetooth 5.0 to an external receiver connected to a laptop.

#### Intravesical pressure IAP measurement (IAP_ivp_)

Following bladder emptying under anesthesia, a latex catheter was inserted transurethrally and connected to a peristaltic pump for saline infusion. Pressure data were captured at 100 Hz using a PowerLab digital system (PowerLab 8/30, AD Instruments, Colorado Springs, CO), with the symphysis pubis serving as the zero-reference point for all measurements, regardless of the animal’s position [16].

#### Direct intraperitoneal IAP measurement IAP_dir_

A multiple-hole catheter was placed intraperitoneally during a small midline laparotomy, positioned caudally to the stomach, and connected to a pressure transducer. Prior to measurements, the catheter was flushed with saline to ensure patency. The pressure transducer was also connected to the PowerLab digital system.

### Experimental protocol

The pigs were positioned in a supine state to standardize the measurement setup. Initial baseline IAP was recorded to provide a reference for subsequent measurements. IAP was incrementally increased using a controlled infusion of carbon dioxide into the peritoneal cavity. Specific pressure targets set for the study were 10, 20, 30, and 40 mmHg. The actual pressure reached was verified using both the direct intraperitoneal catheter (IAP_dir_) and the barosensor **connected** to the inflator. At each target IAP level, there was a **holding up period to permit** the pressure to stabilize into a plateau. This stabilization period was crucial to ensure that the readings were consistent and reflective of a steady state. Following stabilization, IAP measurements were recorded continuously for 5 min to capture any fluctuations and to ensure the accuracy of the data. After recording at one IAP level, the pressure was gradually **diminished to the following** lower preset level or back to baseline. This stepwise **decrease** was carefully managed to avoid rapid changes that could affect physiological responses. Measurements were repeated at each level to assess the reproducibility and reliability of the data. After completing all planned observations and data recordings, the experiment was concluded with the humane euthanasia of the animals. This was carried out by first deepening the anesthesia to ensure no discomfort to the animals, followed by administering an additional dose of 4 mg of Pancuronium Bromide. Subsequently, euthanasia was achieved by injecting 60–100 mL of potassium chloride, effectively inducing cardiac arrest in a controlled and ethical manner.

### Data acquisition and data analysis

Intra-abdominal pressure measurements from the direct intraperitoneal catheter (IAP_dir_) and the intravesical catheter (IAP_ivp_) were acquired using a multimodal monitor (PowerLab 8/30, AD Instruments, Colorado Springs, CO). This equipment was connected to a computer that enabled real-time data capture via a Local Area Network (LAN). Simultaneously, data from the PressureDOT device (IAP_dot_) were wirelessly transmitted to an external receiver and recorded onto a **memory card at a frequency of 0.2 Hz** via a serial port. To ensure the integrity of the data comparisons, the internal clocks of the three computers used for data acquisition were synchronized at the start of the data collection process. Subsequent to data acquisition, time-synchronized IAP readings from the various devices were analyzed offline using dedicated software (Excel, Microsoft Corporation, Seattle, USA). Each data set, referred to as a triplet, was recorded for individual animals, and pressure-time correlation graphs were generated to visually assess the dynamic changes in IAP.

Given the continuous nature of the data collection, we were able to perform detailed comparative analyses between the different IAP measurement modalities for each subject. Summarized data across all subjects were aggregated according to IAP_dir_ levels, facilitating a comprehensive comparison of correlations between IAP_dot_, IAP_ivp_, and IAP_dir_. The IAP_dir_ measurement was considered the reference standard, representing the true pressure within the abdominal cavity. **Disparities** between the measurements obtained from IAP_dot_ and IAP_ivp_ relative to IAP_dir_ were methodically analyzed at set pressure increments of 5, 10, 15, 20, and 30 mmHg. All intra-abdominal pressure values were uniformly expressed in millimeters of mercury (mmHg), standardizing the data for analysis and reporting.

### Statistical analysis

Results are reported as mean ± standard deviation (SD) to quantify the variability and central tendency of the data. The degree of linear correlation between the different intra-abdominal pressure (IAP) measurement methods was evaluated using Pearson correlation coefficients, and the strength of this association was further quantified by calculating the coefficient of determination (R² values). Statistical significance was assessed using a two-tailed t-test, with a p-value of less than 0.05 considered to indicate statistically significant differences between measurement methods.

## Results

**Fig. 3:**
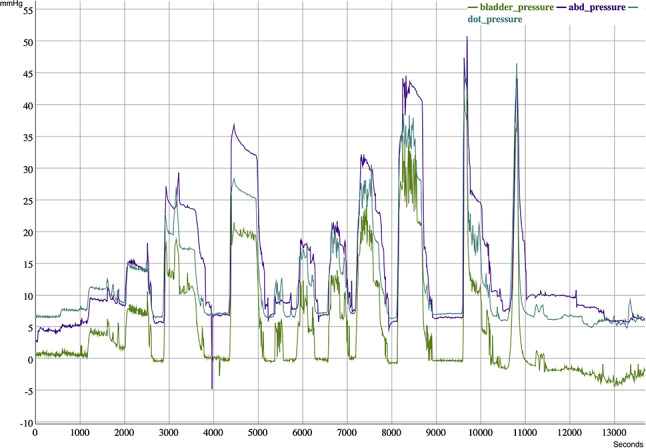
Continuous data depicting intraabdominal pressure measurements from various devices. The purple line represents direct IAP_dir_ measured by an intraperitoneal catheter; the green line represents IAP_ivp_ measured from an intravesical catheter; and the blue line represents IAP_dot_ measured by a capsular pressure device. The figure illustrates the correlation and variance between the three different measuring tools across different pressure levels

The PressureDOT device was successfully inserted transesophageally into all pigs, with placement confirmed via fluoroscopy. In total, 53,065,487 intraabdominal pressure (IAP) measurement triplets were collected to assess the agreement between the different measurement devices. Due to pressure fluctuations or failure to maintain a stable plateau, 238 triplets (comprising 714 individual IAP measurements) were excluded from analysis in the supine position. Additional exclusions occurred during the inflation phase due to instability in pressure levels. Once the IAP measured directly from the peritoneal cavity (IAP_dir_) stabilized at predefined target levels of 5, 10, 15, 20, and 30 mmHg, systematic data recording commenced for IAP_dot_, IAP_ivp_, and IAP_dir_. These measurements were recorded in triplets and illustrated in Fig. 3.

**Fig. 4:**
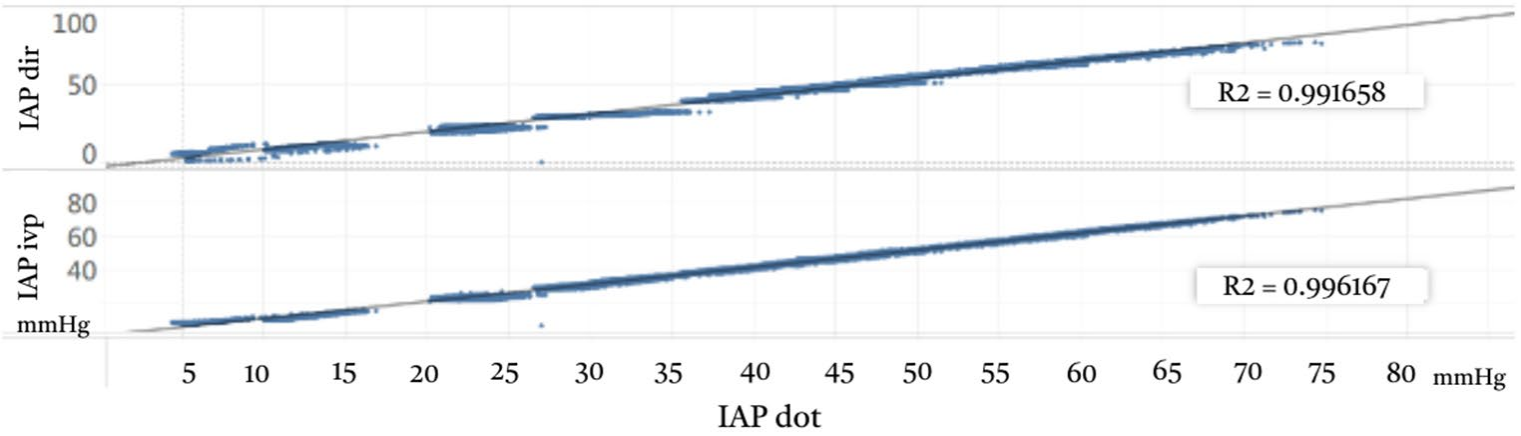
Examination of Correlation between Different IAP Measurement Methods (**A**) IAP_dot_ versus IAP_dir_ showed a strong correlation, with an R² value of 0.9916, indicating excellent agreement between these measurement routes. (**B**) IAP_dot_ versus IAP_ivp_ also demonstrated a strong correlation, with an R² value of 0.9961, underscoring the reliability of the capsular sensor relative to traditional methods

The triplets data was were analyzed to assess the correlation between the intraabdominal pressure measurements obtained via IAP_dot_, IAP_ivp_, and IAP_dir_. The results of these correlation analyses are illustrated in Fig. 4.

Comparative Analysis Across Different Pressure Levels:

All measurement triplets from various pigs were pooled to perform a detailed comparative analysis. At baseline, where the IAP_dir_ was set at 0 mmHg, the mean IAP_dot_ was measured at 0.54 ± 1.11 mmHg, while the mean IAP_ivp_ recorded was 6.02 ± 0.67 mmHg, suggesting initial variance among the devices. During the inflation phase, where IAP_dir_ was maintained at incremental pressure levels, comparative data were gathered:

At a maintained IAP_dir_ of 5 mmHg, the mean values recorded were 3.51 ± 4.18 mmHg for IAP_dot_ and 6.36 ± 3.03 mmHg for IAP_ivp_. At 10 mmHg of IAP_dir_, mean IAP_dot_ was 10.60 ± 4.35 mmHg compared to IAP_ivp_ at 11.71 ± 4.71 mmHg. At 15 mmHg, mean IAP_dot_ recorded was 12.28 ± 4.48 mmHg versus IAP_ivp_ at 9.42 ± 6.32 mmHg. At 20 mmHg, the measurements were 19.10 ± 4.05 mmHg for IAP_dot_ and 22.80 ± 3.95 mmHg for IAP_ivp_. Finally, at 30 mmHg, mean IAP_dot_ was 28.21 ± 4.90 mmHg compared to 32.87 ± 3.86 mmHg for IAP_ivp_. This analysis not only provided insights into the correlation between devices but also allowed for evaluation of consistency and precision across different pressure settings. The results, which highlight the agreement and precision between the measurement techniques, are presented in Table 1.

**Table 1:** The comparison between pressure levels measured by the capsular sensor and intravesical sensor was conducted at various intraabdominal pressure levels

	IAP_dot_ (mean, (SD) mmHg)	IAP_ivp_ (mean, (SD) mmHg)	*P* value
IAP_dir_ = 0	0.55 (1.11)	6.02 (0.67)	< 0.05
IAP_dir_ = 5	3.51 (4.18)	6.36 (3.03)	< 0.05
IAP_dir_ = 10	10.60 (4.35)	11.71 (4.71)	< 0.05
IAP_dir_ = 15	12.28 (4.48)	9.42 (6.32)	< 0.05
IAP_dir_ = 20	19.10 (4.05)	22.80 (3.95)	< 0.05
IAP_dir_ = 30	28.21 (4.90)	32.87 (3.85)	< 0.05
R^2^	0.9241	0.9168	

IAP_dir_ : Intraabdominal pressure measured by direct peritoneal cathter

IAP_dot_ : Intraabdominal pressure measured by capsular pressure device

IAP_ivp_ : Intraabdominal pressure measured by intravesical catheter

SD: standard deviation

**Fig. 5:**
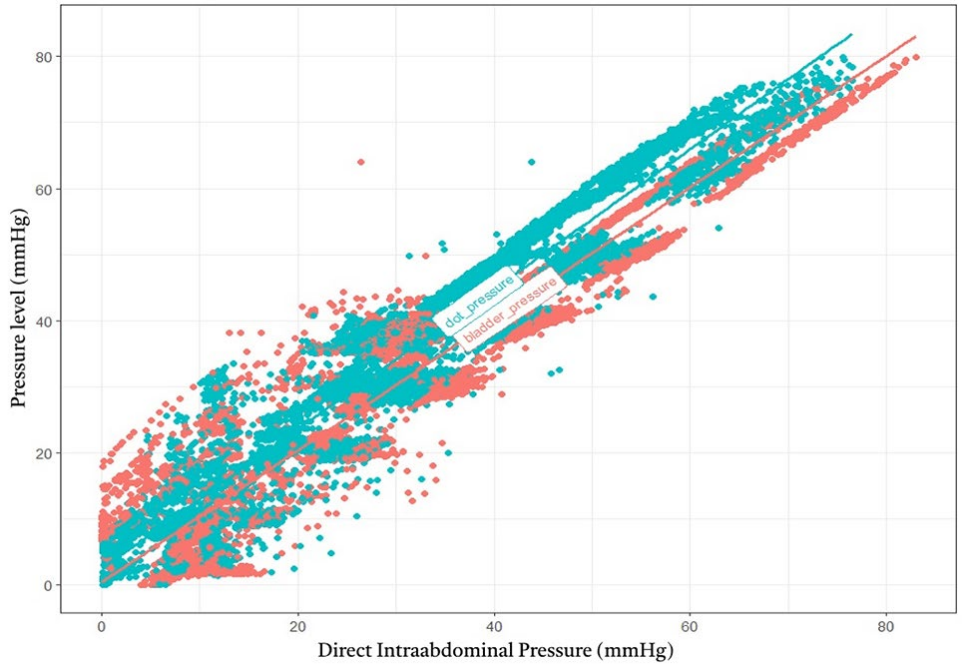
Correlation Dot Plot Between Different Intraabdominal Pressure Measurement Methods This figure visually summarizes the pooled pressure comparisons across different measurement routes. There is excellent correlation between IAP_dot_ and IAP_dir_ with an R² value of 0.9241. There is also strong correlation between IAP_ivp_ and IAP_dir_, with an R² value of 0.9168

The correlation between IAP_dot_ and IAP_dir_ yielded an R² value of 0.9241, indicating excellent agreement. Similarly, the correlation between IAP_ivp_ and IAP_dir_ demonstrated robustness with an R² value of 0.9168. These findings suggest that both alternative measurement methods closely align with the direct intraperitoneal measurements. The summarized correlation dot plot is depicted in Fig. 5.

## Discussion

This study contributes significant insights into the utility of a novel capsular device for continuous IAP monitoring. The device’s capability to accurately measure and wirelessly transmit IAP readings over extended periods was clearly demonstrated. Notably, the capsular device (IAP_dot_) maintained continuous monitoring for several days, presenting a substantial advancement over traditional methods. The accuracy of the capsular device was affirmed by its excellent correlation with the direct intraperitoneal measurement (IAP_dir_), which is considered a proxy for the true intraabdominal pressure. The correlation coefficient (R²) of 0.9241 between IAP_dot_ and IAP_dir_ underscores this point. Similarly, the correlation between the capsular device and the intravesical measurement (IAP_ivp_), the current standard method, was also robust (R² = 0.9168). These correlations highlight the capsular device’s potential to replace or complement existing techniques.

The capsular device’s closer agreement with IAP_dir_ than with IAP_ivp_ may be attributed to anatomical and physiological factors. The urinary bladder, where IAP_ivp_ is measured, resides in the retroperitoneal space and may exhibit slight pressure gradient differences from the intraperitoneal cavity where true IAP is more directly assessed. Thus, IAP_dot_ provides a more accurate reflection of the intraperitoneal environment compared to IAP_ivp_. Advancements in biotechnology and electrical design have enabled the development of wireless devices that are both power-efficient and capable of high-frequency data transmission. This allows for real-time, continuous monitoring of IAP, which is crucial for timely therapeutic decisions. The continuous data stream offered by such devices ensures immediate access to IAP levels via external receivers by healthcare providers, enhancing patient monitoring and management.

Beyond real-time, another benefits the wireless capsular sensor offered is to provide continuous IAP level. Continuous monitoring of IAP is invaluable in critical care, improving the understanding of abdominal dynamics and empowering healthcare providers to intervene proactively, potentially saving lives and improving the overall quality of patient care [17]. By integrating continuous IAP data with mean arterial pressure (MAP) readings, it is feasible to develop a continuous abdominal perfusion pressure (APP) metric. Such integration could provide immediate evaluations of visceral perfusion, maintaining it within optimal levels to prevent end-organ damage [18]. 

While traditional devices provide accurate, even if intermittent, IAP measurements, they fall short in delivering continuous data necessary for effective end-organ perfusion monitoring and dynamic clinical decision-making. The introduction of devices capable of continuous IAP monitoring promises not only to enhance patient recovery but also to reduce healthcare costs by preventing complications associated with fluctuating IAP levels [19–23]. Ensuring that pressures remain within safe ranges could mitigate risks such as organ dysfunction and respiratory compromise, significantly improving outcomes in critically ill patients [17, 24, 25]. Continuous IAP monitoring facilitates the development of personalized treatment plans, allowing clinicians to tailor interventions based on dynamic pressure changes and address each patient’s unique needs. This level of customization is vital in critical care settings where standard protocols may be insufficient.

Intra-abdominal hypertension (IAH) and abdominal compartment syndrome (ACS), characterized by sustained increases in IAP, are associated with high morbidity and mortality rates if left untreated. Continuous IAP monitoring enables early identification of patients at risk for ACS, allowing for timely intervention and prevention. This continuous data stream provides a comprehensive assessment of intra-abdominal dynamics, revealing trends and patterns that might be missed with intermittent measurements. The importance of early detection and intervention is particularly significant in trauma and surgical patients.

Compared to IAP measurement via urinary catheter, the capsular sensor demonstrates several advantages. First, it enables automatic pressure detection without inflating the intravesical space or instilling fluid back into the bladder. This preserves the urinary system’s sterile environment, reducing the risk of retrograde urinary tract infection and regurgitation. Second, the capsular sensor automates IAP monitoring, reducing the workload in critical care settings—a significant benefit amidst the ongoing shortage of healthcare providers. Third, it eliminates the issue of bladder compliance variance, which can affect IAP_ivp_ measurements taken via urinary catheter due to interstitial changes or bladder inflammation [26, 27]. Furthermore, the capsular device allows for accurate IAP monitoring regardless of body position or abdominal muscle contracture, overcoming the limitations of traditional methods that require patients to remain supine and at rest [28]. Finally, the capsular sensor enables IAP monitoring in patients without a Foley catheter, potentially extending its use to outpatient settings.

This study presents a novel capsular monitoring system for IAP detection, demonstrating the feasibility and effectiveness of capsular pressure sensors for continuous, real-time IAP monitoring. Despite the advantages highlighted in this study, several limitations warrant acknowledgment. First, the current design necessitates capsule delivery by a healthcare provider for unconscious patients, potentially hindering widespread implementation. Specialized devices for automatic capsule insertion into the GI tract could address this limitation. Second, while our study shows a promising correlation between IAP_dot_, IAP_ivp_ and IAP_dir_, further animal studies and clinical trials are needed to validate these findings and establish broader evidence supporting this method.

## Conclusion

This study introduces a capsular pressure monitoring device as an innovative and alternative method for IAP assessment. The continuous and accurate IAP monitoring facilitated by capsular sensors represents a paradigm shift, offering clinicians and researchers a more comprehensive understanding of visceral perfusion dynamics. This technology has the potential to significantly impact clinical practice, enabling timely interventions based on real-time, continuous monitoring data, ultimately leading to improved patient outcomes. *By combining MAP and IAP, we aim to establish a reliable and continuous APP metric*, ensuring real-time evaluation and maintenance of visceral perfusion within optimal and standardized levels.

## Data Availability

The dataset is not publicly available due to restrictions in the data sharing agreements with the Chang Gung Memorial Hospital Institutional Review Board (IRB). The partial dataset was available by the request to corresponding authors under academic purpose.
